# Identification and characterization of phage protein and its activity against two strains of multidrug-resistant *Pseudomonas aeruginosa*

**DOI:** 10.1038/s41598-019-50030-5

**Published:** 2019-09-17

**Authors:** Fairoz Al-Wrafy, Ewa Brzozowska, Sabina Górska, Marek Drab, Magdalena Strus, Andrzej Gamian

**Affiliations:** 1 Department of Applied Microbiology, Faculty of Applied Sciences, Taiz University, 6350 Taiz, Yemen; 2 Hirszfeld Institute of Immunology and Experimental Therapy, Polish Academy of Sciences, 53-114 Wrocław, Poland; 3 Department of Microbiology, Medical College, Jagiellonian University, 31-007 Krakow, Poland

**Keywords:** Bacteriophages, Bacteriology

## Abstract

*Pseudomonas aeruginosa* is an opportunistic pathogen with a capacity to develop antibiotic resistance, which underlies a larger proportion of hospital-acquired infections and higher morbidity and mortality, compared to other bacterial infections. Effective novel approaches for treatment of infections induced by this pathogen are therefore necessary. Phage therapy represents a promising alternative solution to eradicate antibiotic-resistant pathogens. Here, we investigated phage protein efficacy against multi-drug resistant (MDR) *P. aeruginosa* PAR21 and PAR50 strains isolated from diabetic foot ulcer patients. The results obtained using spot assay, zymography, spectrophotometry and scanning electron microscopy at low voltage (SEM-LV) indicate that the phage protein, PA-PP, exerts activity against *P. aeruginosa* PAR50 while having no impact on the PAR21 strain. Using LC-MS-MS/MS and comparative analysis of the peptide molecular mass with the protein sequence database, PA-PP was identified as a member of the serine protease family, a result corroborated by its ability to digest casein. We additionally showed a capacity of PA-PP to digest porin protein on the bacterial outer membrane (OM). Moreover, synergistic activity between PA-PP protein and piperacillin led to higher sensitivity of bacterial cells to this antibiotic. Our collective findings suggest that PA-PP targets porin protein on PAR50 OM, thereby increasing its sensitivity to specific antibiotics. The adverse effects observed on bacterial cells using SEM-LV suggest further roles of this protein that remain to be established.

## Introduction

*Pseudomonas aeruginosa* is an opportunistic pathogen responsible for several acute and chronic infections in humans, including meningitis, abscess, infection of skin, soft tissues, urinary tract, bones and joints and conjunctival erythema in addition to a variety of systemic infections in individuals with genetic diseases as in cystic fibrosis patients (CF), immunocompromised patients, diabetes mellitus patients, and those receiving chemotherapy^1^. Notably, the highest number of mortality cases and lengths of hospital stay have been documented in patients with multi-drug resistant (MDR) *P. aeruginosa* infections^2,3^. The emergence of MDR *P. aeruginosa* strains is attributed to several factors that may be intrinsic and/or acquired^4^. Outer membrane permeability^5^, AmpC lactamases^6^ and membrane efflux pumps (Mex)^7^ are intrinsic resistance mechanisms whereas biofilm formation, swarming motility or other complex adaptations^8^ and those occurring due to genetic transfer and mutations^9^ are acquired resistance mechanisms. Regardless of the mechanism of resistance, the prevalence of MDR strains poses a critical medical problem that necessitates further comprehensive investigation to uncover novel approaches for eradicating these pathogens and associated diseases.

Phages and their components represent a suitable therapeutic solution in view of their ability to target pathogenic bacteria at the site of infection without affecting normal flora and gradual disappearance after the demise of the host in addition to their capacity to influence bacterial biofilms that play roles in the antibiotic resistance^1^. For instance, phages phiIB-PAA2 and PAØ, defined as broad bactericidal and anti-biofilm agents^10,11^, and engineered T7 phage, inhibit biofilm formation and quorum sensing activity for both *P. aeruginosa* and *E. coli*^12^, as well as three phages belong to PB1-like viruses, phiKZ-like viruses and LUZ24-like viruses, showed lytic activity against clinical isolates of MDR *P. aeruginosa*^13^. Nevertheless, the effective clinical application of phage therapy is difficult due to the ever-changing nature of phages and their ability to transfer genes between bacteria and potentially interact with the human immune system^14,15^. The capacity of bacteria to develop resistance to phages^16,17^ and release bacterial components after phage infection, such as endotoxins that cause septicemia, a phenomenon known as the Jarisch–Herxheimer reaction^18^, can also represent additional problems in phage therapy.

Bacteriolysis by phage occurs either during the adsorption stage when a large number of phage particles attach to the same bacterial cell (lysis from outside) or at the end of the lytic cycle by disruption of the cell wall via the endolysin- holin- spanin systems or single protein lysis system (lysis from inside)^19,20^. During the infection cycle, the phage produces several proteins that play important roles in its multiplication and release progeny phages from the infected bacterium, triggering bacterial cell death. Importantly, the undesired features that accompany the use of the whole phage as a therapeutic agent can be avoided by using phage proteins instead of the phage itself^1^. The most notable phage products for therapeutic consideration are phage-encoded peptidoglycan hydrolases (PGH) i.e. endolysins, polysaccharide depolymerases and holin (cell membrane-disrupting protein)^21^, in addition to those involved in cell wall synthesis inhibition^20^. These proteins exhibit high efficacy against bacterial cells, either killing them or leading to intrinsic changes in their structures, thereby facilitating lysis by other factors. For instance, *Pseudomonas* phage lysins KZ144 and EL188 bind peptidoglycan of *P. aeruginosa*^22^, and alginate lyase or alginase degrades alginate capsular polysaccharide to facilitate phage penetration^23^ and migration within the biofilm of *P. aeruginosa*^24^. Degradation of alginates in pseudomonal CF strains by phage PT-6 alginase is additionally reported to accelerate phagocytic uptake of bacteria and disrupt microbial growth in biofilms^25^. Recently, phage display technology classified as a powerful technique in the screening of peptide with high affinity and selectivity, where the phage display derived products can play a significant role in the diagnosis and treatment of disease^26,27^. For example, pVIII fusion proteins isolated from phage GQTTLTTS and phage VQTVQIGSD were selected from the f8/8 and f8/9 landscape phage library against *Staphylococcus aureus* in and *Vibrio parahaemolyticus* in high throughput and selectivity^28,29^.

Combined treatment with phage and antibiotic may present a critical step in improving antibiotic efficacy through enhancing drug delivery to specific cells and increasing local drug concentrations^30^. Earlier studies have reported higher efficacy of combination treatment than either agent alone in terms of reducing bacterial levels in the lung, liver, kidney, spleen, and blood of mice. In addition, neutrophil infiltration and inflammatory cytokine counts were reduced, which were attributed to restoration of the functionality of overused antibiotics by phage enzymes^21^. Consistent with these findings, a combination of phage and tobramycin led to significant reduction of the emergence of antibiotic- and phage-resistant cells in both *E. coli* and *P. aeruginosa* biofilms^31^. Similar results were obtained upon co-treatment of *P. aeruginosa* EE with phage and ciprofloxacin^32^. In another recent report, utilization of a specific phage against MDR *P. aeruginosa* triggered changes in the efflux pump mechanism critical in antibiotic resistance, which led to increased sensitivity to several antibiotics^33^.

The current study was designed to investigate the efficiency of a phage protein, PA-PP, against MDR *P. aeruginosa* PAR21 and PAR50 strains isolated from diabetic foot ulcer patients. We further focused on identifying the specific PA-PP protein receptor on *P. aeruginosa* and evaluating its activity in conjunction with antibiotics.

## Results

### Sensitivity test for antibiotics and bacteriophage

The *P. aeruginosa* PAR21 and PAR50 strains used in this study demonstrated variance in response to antibiotics, with greater resistance of PAR50 than PAR21. Among the 13 antibiotics examined, PAR50 was resistant to piperacillin, ticarcillin-clavulanic acid, ceftriaxone, amikacin, gentamicin and tobramycin whereas PAR21 was resistant to ceftriaxone only, as shown in Supplementary Table S1. In contrast, the PAR21 strain was resistant to infection by phage whereas PAR50 appeared highly sensitive, as evident from the halo zone observed with the spot assay on the agar plate with or without plaques in the case of *P. aeruginosa* PAR50 but not PAR21, signifying efficacy of the phage against the PAR50 strain (Supplementary Fig. S1).

### Purification and identification of PA-PP protein

**Figure 1:**
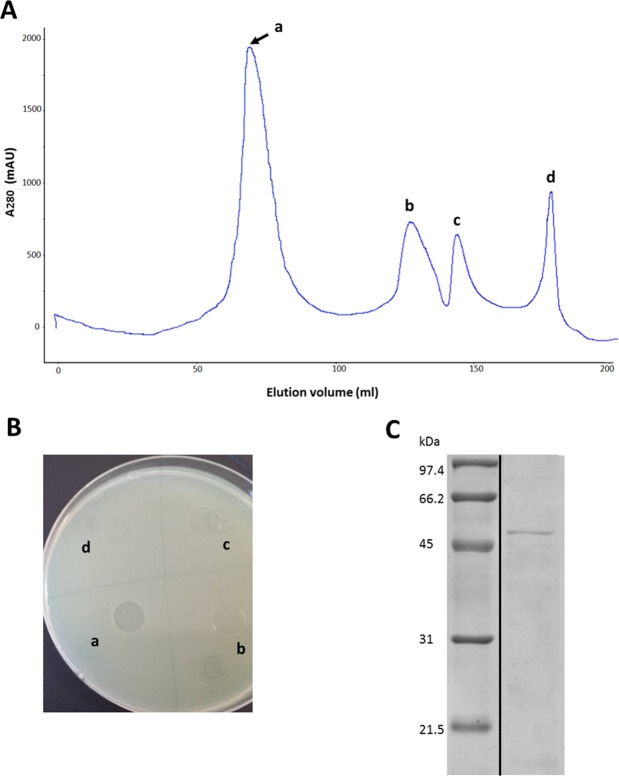
Purification of PA-PP protein. (**A**) Elution profile of purified PA-PP using a HW-55S column with 0.06 M phosphate buffer, pH 7.2, as eluent. Four fractions were collected: a, b, c and d. (**B**) Spot assay on the agar plate with *P. aeruginosa* PAR50. Among the four fractions, only one (a) showed efficacy against *P. aeruginosa* PAR50 based on a transparent spot on the agar plate. (**C**) SDS-PADE of PA-PP using a 12.5% polyacrylamide gel followed by staining with CBB. The molecular mass of protein was between 45 and 66 kDa. Full-length uncropped gel is presented in Supplementary Fig. S2.

**Figure 2:**
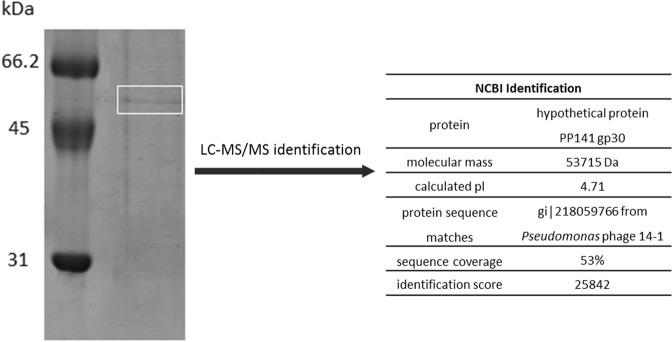
Identification of PA-PP protein. The protein was separated via SDS-PAGE, and the target band in the white box excised and subjected to LC-MS/MS. Peptide molecular masses were compared with the protein sequence database (NCBI, UniProt database). The details of the comparative analysis are presented in the table on the right. Full-length gel is presented in Supplementary Fig. S3.

Isolation of PA-PP protein from phage particles with 0.1 N HCl, followed by purification via gel filtration chromatography on a HW-55S column with 0.06 M phosphate buffer generated four fractions designated a, b, c and d (Fig. 1A). All fractions were subjected to the spot assay to ascertain activity against *P. aeruginosa* strains. Fraction (a) showed clear efficacy against *P. aeruginosa* PAR50 compared to the other fractions (Fig. 1B). SDS-PAGE (12.5%) of the target protein (fraction a) and Coomassie Brilliant Blue-R250 (CBB-R250) staining revealed a band with a molecular mass between 45 and 66 kDa (Fig. 1C). For identification of PA-PP, the band was excised from the gel, treated with trypsin, and analyzed via LC-MS/MS, followed by comparative evaluation of peptide masses in the UniProt database (NCBI) using the Mascot program. Consequently, PA-PP was identified as a hypothetical protein, PP141_gp30, with a molecular mass 53.7 kDa, pI 4.71, identification score of 25842 and sequence coverage of 53% (Fig. 2). Further search in the Mascot engine using comparative analysis of peptide sequences revealed that PA-PP protein belongs to the serine proteases family. The protein sequence is included in Supplementary Fig. S4.

### Zymography

**Figure 3:**
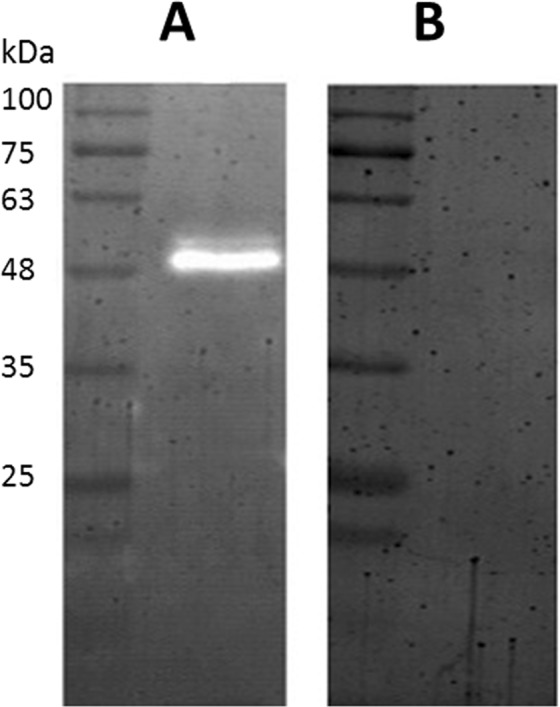
Electrophoresis of PA-PP protein on a 12.5% gel containing 0.1% bacterial cells under non-reducing conditions and renaturation using Triton X-100 buffer. The influence of PA-PP against PAR50 is evident based on the transparent band on gel (**A**). PA-PP had no influence on PAR21 strain on the gel (**B**). Full-length gels, A and B, are included in Supplementary Fig. S5.

The activity of PA-PP against *P. aeruginosa* strains was comprehensively evaluated. The spot assay was applied as the first step to verify protein activity, as shown in Fig. 1B. Zymography further confirmed activity of the protein against *P. aeruginosa* PAR50. PA-PP protein was loaded under non-reducing conditions on a 12.5% polyacrylamide gel containing 0.1% (w/v) *P. aeruginosa* cells, which was renatured by incubating in renaturation buffer at 37 °C for 16 h, followed by staining with CBB-R250 to distinguish the interacting areas on the blue background of the gel. The interaction area between bacteria and PA-PP appeared as a transparent band on the gel with the PAR50 strain, as presented in Fig. 3A. However, no reaction between PA-PP and PAR21 was observed (Fig. 3B).

### Spectrophotometric assay

**Figure 4:**
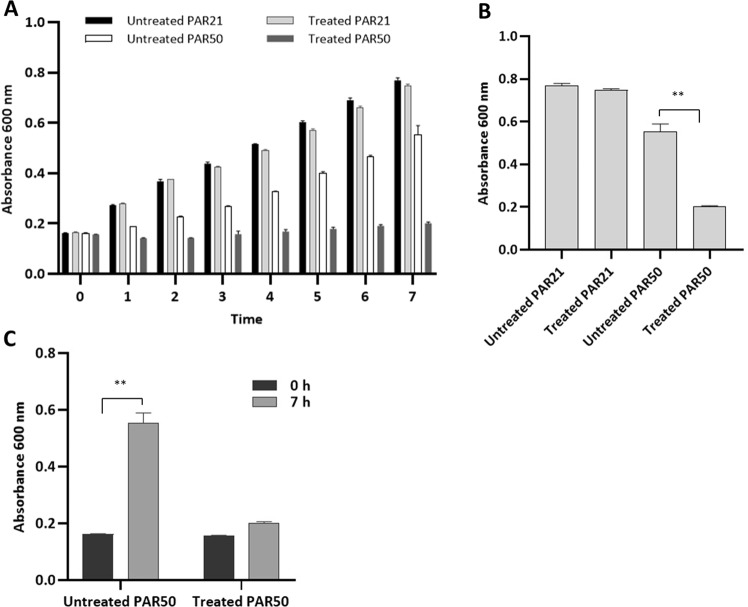
Evaluation of the efficacy of PA-PP protein against growth of *P. aeruginosa* strains PAR50 and PAR21. (**A**) Spectrophotometric absorbance for both strains with/without PA-PP (100 µg/ml) at 600 nm every hour for 7 h. PAR50 treated with PA-PP protein displayed inhibition of growth, compared to untreated strain, which showed increased growth with time. No significant differences were evident in the growth of PAR21 strains with and without protein. (**B**) Effect of PA-PP on each bacterial strain after 7 h of incubation. (**C**) Comparison of growth of treated PAR50 at 0 h and 7 h. Values are presented as means ± SEM from three independent measurements, **P < 0.01.

**Figure 5:**
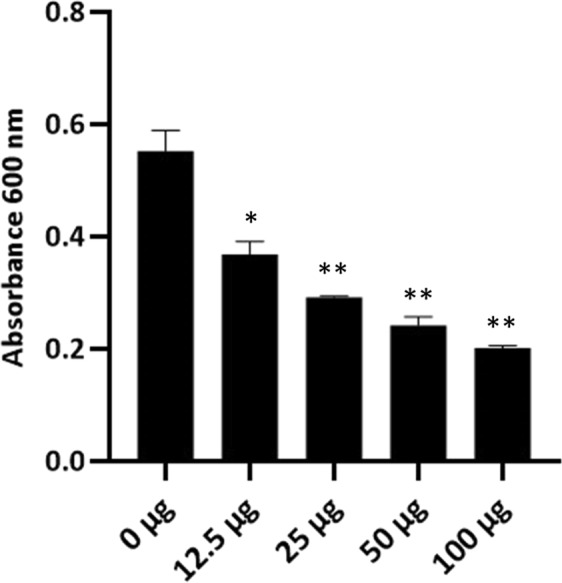
Effects of different concentrations of PA-PP on *P. aeruginosa* PAR50 growth. The PAR50 bacterial strain was incubated with different concentrations of PA-PP protein (0, 12.5, 25, 50 and 100 µg/ml) at 37 °C and absorbance read at 600 nm after 7 h. Lowest bacterial growth was evident at high concentrations of PA-PP (100 µg/ml). Values are expressed as means ± SEM from three independent measurements. *P < 0.05 and **P < 0.01.

Evaluation of PA-PP protein efficacy against *P. aeruginosa* PAR21 and PAR50 via gradual measurement of absorbance at 600 nm every hour for 7 h using a mixture of bacterial cells and PA-PP (100 µg/ml) disclosed that growth of PAR50 did not progress with time relative to that of treated PAR21, which showed continued growth along with untreated PAR21 (Fig. 4A). Comparison of growth of both treated and untreated PAR50 after incubation for 7 h at 37 °C further confirmed notably decreased growth of treated PAR50 (*P* < 0.01) (Fig. 4B). Comparison of treated PAR50 growth at the start (0 h) and end of the experiment (after 7 h) disclosed no remarkable progression (Fig. 4C) with >90% inhibition of bacterial growth. Experiments with different dilutions of PA-PP (0, 12.5, 25, 50 and 100 µg/ml) showed a greater impact with increasing protein concentration on the PAR50 strain, with highly significant differences in bacterial growth at each concentration examined (*P* < 0.05 and *P* < 0.01) (Fig. 5).

### Assay of enzymatic activity

**Figure 6:**
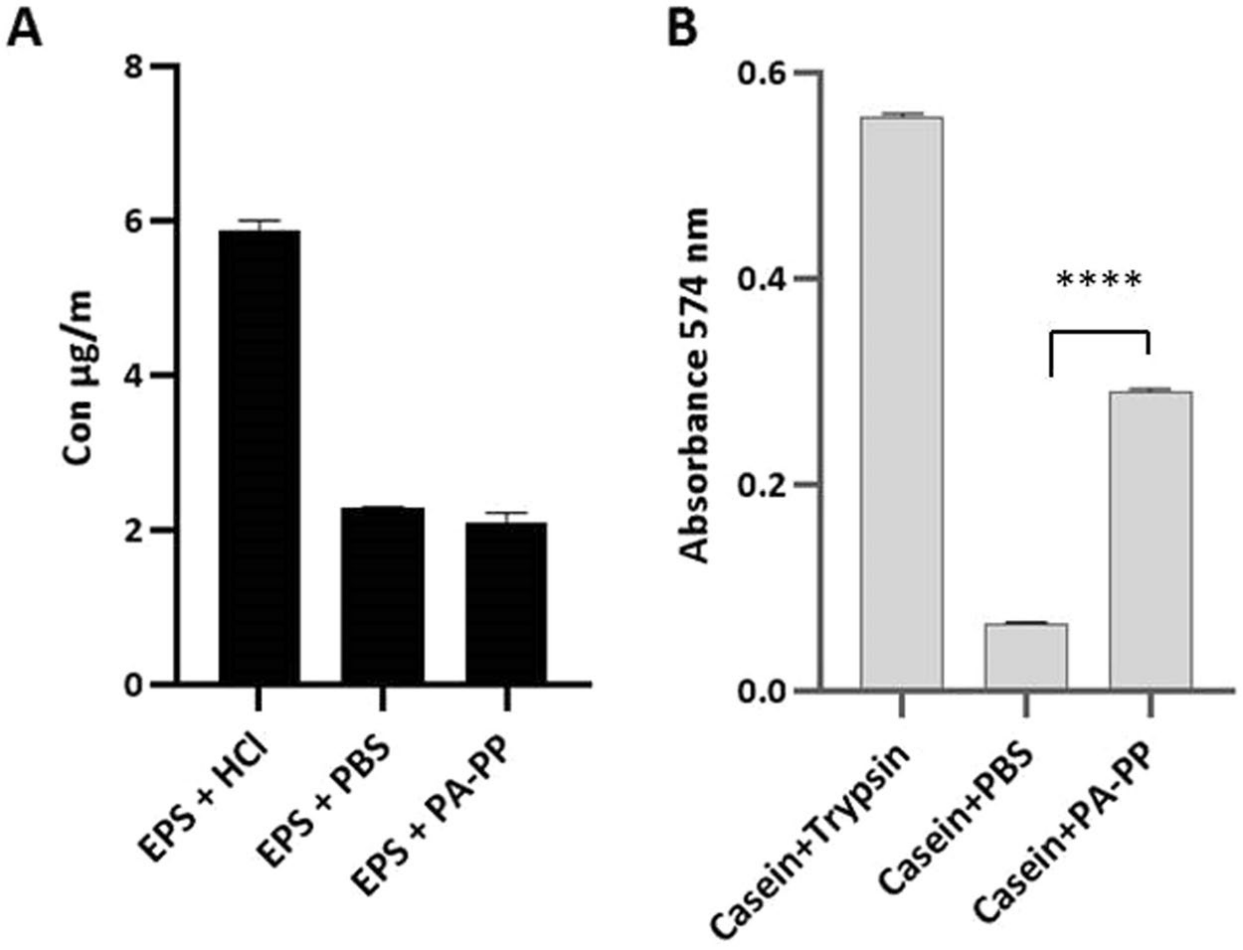
Evaluation of the enzymatic activity of PA-PP protein. (**A**) PA-PP protein-mediated digestion of EPS of *P. aeruginosa* PAR50. EPS with 10 M HCl and EPS with PBS were used as the positive and negative controls, respectively. Our data show that the PA-PP protein does not affect bacterial EPS. (**B**) Proteolytic activity of PA-PP against commercial casein. Resorufin-labeled casein with trypsin and casein with PBS were used as positive and negative controls, respectively. Resorufin-labeled peptides released from digested casein were measured to determine the degradation ability of PA-PP. Values are expressed as means ± SEM from three independent measurements, ****P < 0.0001.

Bacterial EPS and commercial casein were used as substrates to evaluate the enzymatic activity of PA-PP. Determination of the ability of PA-PP to degrade PAR50 EPS via the Nelson-Somogyi method revealed no effect of the protein. As shown in Fig. 6A, no differences were evident in the sugar contents among both EPS samples exposed to PA-PP and PBS, while HCl hydrolysis of EPS resulted in a higher concentration of sugars. The proteolytic activity of PA-PP was estimated using resorufin-labeled casein as a substrate. The ability of PA-PP to degrade casein was determined by measuring the absorbance of resorufin-labeled peptides released from degradation of casein at 574 nm. A significant level of resorufin-labeled peptides was released from casein digested with PA-PP, compared with control (*P* < 0.0001), suggesting the capability of PA-PP to utilize casein as substrate (Fig. 6B).

### Scanning electron microscopy at low voltage (LV-SEM)

**Figure 7:**
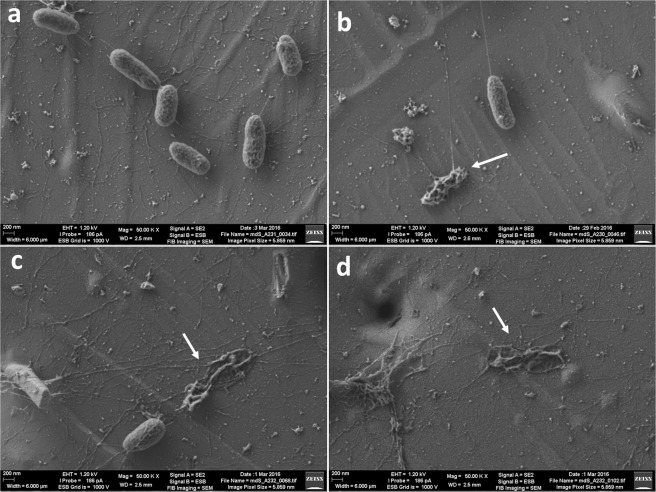
Low voltage scanning electron microscopy (LV-SEM) of *P. aeruginosa* PAR50 with or without PA-PP protein. The effects of PA-PP on the bacterial cells of *P. aeruginosa* PAR50 are shown in images (**b**, **c** and **d**) (the arrows highlight bacterial cell death, i.e., “ghosts or remnants”). Image (**a**) represent healthy bacterial cells not exposed to PA-PP protein.

Incubation of PA-PP with bacterial colonies on agar plates and subsequent LV-SEM revealed a significant impact on *P. aeruginosa* PAR50, compared with control samples. The protein caused phenotypic changes in cells of this strain, which were visualized using LV-SEM as shortening of bacterial cells (owing to defects in the ratio of the length to width axis) as well as collapse of bacterial bodies. Our data indicate that PA-PP is able to stimulate structural alterations at the whole bacterial level, as illustrated in Fig. 7. Notably, these defects were not observed in PAR21 cells exposed to the PA-PP protein Supplementary Fig. S6.

### Detection of phage receptor on the bacterial cell surface

**Figure 8:**
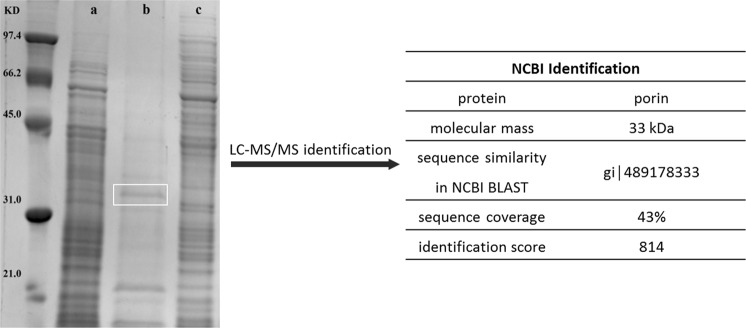
Identification of PA-PP-interacting OM proteins of *P. aeruginosa* PAR50. OM proteins isolated from PA-PP-treated and untreated PAR50 strains were analyzed via SDS-PAGE. Lanes (a and b) represent all OM proteins and some OM proteins (porin proteins) respectively, isolated from untreated *P. aeruginosa* PAR50. Lane c is OM proteins isolated from treated *P. aeruginosa* PAR50. The band in the white box disappeared from lane c. This band was excised from the gel and analyzed using LC-MS/MS. Peptide molecular masses were compared with protein sequences in the UniProt database (NCBI). Details of the identification are presented in the table on the right. Full-length gel is presented in Supplementary Fig. S7.

The PA-PP receptor was determined on the surface of *P. aeruginosa* PAR50 in both EPS and OM proteins. To identify the receptor, EPS was isolated and interactions with PA-PP examined using zymography and the Nelson-Somogyi method. In both experiments, PA-PP exerted no activity against EPS (Fig. 6A). To reveal the specific binder of PA-PP among the OM proteins, bacteria were incubated with PA-PP at 37 °C for 18–24 h with shaking, using untreated PAR50 strain as a control. OM proteins were isolated from treated and untreated PAR50 using the SDS extraction method and analyzed via SDS-PAGE to compare protein contents between the samples. We observed differences in the OM protein contents between untreated (Fig. 8, lanes a and b) and treated (Fig. 8, lane c) *P. aeruginosa* PAR50. A 31–45 kDa band that appeared in the untreated lane disappeared in the treated lane. This band was excised and subjected to LC-MS/MS, followed by comparison of molecular masses with the protein sequence database (NCBI, UniProt database) using the MASCOT program, which led to its identification as porin protein with a molecular mass of 33 kDa, identification score of 814 and sequence coverage of 43% (Fig. 8). The protein sequence is included in Supplementary Fig. S8.

### Evaluation of the impact of PA-PP protein on antibiotic activity

Application of antibiotic discs on an agar plate containing *P. aeruginosa* PAR50 previously treated with PA-PP protein demonstrated a positive impact of the protein on the activity of specific antibiotics where the inhibition zone diameters appeared larger than for those applied on the agar plate with untreated *P. aeruginosa* PAR50 (Table 1). Piperacillin demonstrated higher efficacy against the treated (with 22 mm in diameter of inhibition zone) than untreated strain (with 14 mm in diameter of inhibition zone) whereas a slight change in the actions of ticarcillin-clavulanic acid and amikacin was observed against the treated strain (Supplementary Fig. S9).

**Table 1:** Inhibition zone diameters of antibiotic against untreated and PA-PP-treated *P. aeruginosa* PAR50. R. Resistant, I. Intermediate and S. Susceptible.

Antimicrobial agent	Disk content	Zone diameter according to the criteria published CLSI			Zone diameter [mm]			
		R	I	S	Untreated		Treated	
Piperacillin	PRL_100_	≤14	15–20	≥21	14	**R**	22	**S**
Ticarcillin-clavulanic acid	TIM_85_	≤15	16–23	≥24	14	**R**	16	**I**
Gentamicin	CN_10_	≤12	13–14	≥15	<12	**R**	<12	**R**
Amikacin	AK_30_	≤14	15–16	≥17	14	**R**	16	**I**
Tobramycin	TOB_10_	≤12	13–14	≥15	<12	**R**	<12	**R**

## Discussion

Phages have attracted increasing research interest as they possess components with lytic activity against bacterial cells or their constituents, such as cell wall^21^ or biofilm matrix^11,34^. Phages are additionally reported to act in collaboration with antibiotics^32^. In this study, we isolated a phage protein, PA-PP, with high efficacy against MDR *P. aeruginosa* PAR50 isolated from diabetic foot ulcer patients. This strain is resistant to a range of antibiotics, including piperacillin, ticarcillin-clavulanic acid, ceftriaxone, amikacin, gentamicin, and tobramycin. *P. aeruginosa* PAR21 also isolated from diabetic foot ulcer patients, was more sensitive to these antibiotics than PAR50, but not affected by phage and its protein. Other studies are in agreement with our findings, several phage proteins were identified with their activity against *P. aeruginosa*, for instance, *Pseudomonas* phages lysins KZ144 and EL188 with peptidoglycan hydrolysis role^22^, phage PT-6 alginase that reduce the viscosity of alginate, thereby facilitating phage migration through *P. aeruginosa* biofilms^25^, and *Pseudomonas* phage LKA1 depolymerase that cause biofilm degradation^35^. On the other hand, the results obtained in experiments either *in vivo* or *in vitro* proved phage efficiency against *P. aeruginosa* infection, where the injection of phage into mice with septicemia caused by MDR *P. aeruginosa* led to bacterial death and rescued 100% mice with mild infection and 50% moribund mice^36^. A high efficacy has been demonstrated also when the cocktail of phages was applying into ears of patients with chronic otitis caused by MDR *P. aeruginosa*^37^. More recently, intravenous injection of phage cocktail BFC1 against *P. aeruginosa* septicemia developed from acute kidney injury led to negative blood cultures and kidney function recovered after a few days and no unexpected adverse events were observed, that could be related to the application of bacteriophages^38^. Furthermore, high efficacy was reported for phages against MDR *P. aeruginosa* isolated from patients with chronic pulmonary infection^39^, cystic fibrosis patients^40,41^ and other clinical samples^14^. Indeed, the bacteria possess several mechanisms of phage resistance include restriction-modification systems, CRISPR/Cas systems, and abortive infection systems. In addition, mechanisms of phage resistance within biofilms pose via hiding of phage receptors behind barriers consisting of extracellular polymers leading to prevent phage attachment on the bacterial cell^42^. The chemical diversity of these extracellular polymers among bacterial strains could be the product of a phage-mediated frequency-dependent selection^16^. The immunochemical analysis of PAR21 and PAR50 exopolysaccharides showed that each EPS has a different structure (data not shown), this may explain why the activity of phage and its protein was only against PAR50 strain.

The PA-PP activity against *P. aeruginosa* PAR50 was evaluated via spot assay, zymography, spectrophotometry and LV-SEM. All experiments confirmed high efficacy of PA-PP activity against the PAR50 strain. For instance, the emergence of a transparent band on the gel with 0.1% (w/v) PAR50 strain loaded with PA-PP in the zymography profile indicated that interactions between bacterial cells and phage protein led to depletion of bacteria from this area (Fig. 3). Additionally, spectrophotometric monitoring of bacteria incubated with PA-PP protein showed that PAR50 growth was terminated, compared to control and PAR21 strain, which continued growing, analogous to a study showing that a hypothetical protein gp70 of *Pseudomonas* phage 14-1 has strong inhibitory effect on growth on solid medium^43^, furthermore, similar results were found by incubation of MDR *P. aeruginosa* MDR-PA1-5 strain with phage SL2 for 16 h^13^, and incubation of MDR *P. aeruginosa* 2995 strain with phage^44^.

LV-SEM analyses revealed a substantial impact of the purified protein on *P. aeruginosa* PAR50, where phenotypic changes in bacteria were evident, signifying induction of structural defects and deformation at the whole-cell level (Fig. 7). Signals of disintegration within bacterial cells were observed in the bacterial population exposed to the protein but not cells exposed to PBS. The concentration of purified protein was sufficient for generating structural deformations in bacterial cells without the need of additional factors. The degree of structural deformation was substantial and the presence of dead bacterial bodies suggested lethal effects. PA-PP-induced changes in dimensions with relative reduction of the longitudinal axis and expansion of bacterial width indicate that the bacterial cell envelope is targeted by this protein, corroborated by our other findings as discussed below.

To determine the receptor of PA-PP on *P. aeruginosa* surface, exopolysaccharides from both strains (PAR21 and PAR50) were isolated and exposed to protein, using zymography and the Nelson-Somogyi method to evaluate the ability of PA-PP protein to digest EPS. No positive results were obtained in both experiments, indicating that EPS is not a substrate of PA-PP protein, in contrast to data from earlier studies focusing on the efficacy of phage and its components on bacterial EPS^13,34,45^. Nevertheless, the protein had a strong impact on bacterial cells.

LC-MS/MS analysis of PA-PP protein followed by comparative analysis of peptide masses and sequences in the UniProt database (NCBI) using the Mascot search engine identified PA-PP protein as a hypothetical protein PP141_gp30, with a molecular mass 53.7 kDa, identification score of 25842 and sequence coverage of 53% (Fig. 2). A hypothetical protein PP141_gp30 was identified first time as a major building block of the phage particle and is produced by *Pseudomonas* phage 14-1, a PB1-like virus^46^. Further investigation using comparative analysis of peptide sequences in the UniProt database (NCBI) using the Mascot search engine identified PA-PP as one of serine protease family, consistent with its capacity to degrade commercial casein (Fig. 6B). The identification using Mascot search is well known and routinely used, where ɸKZ protease, gp175, isolated from *P. aeruginosa* phage ɸKZ was identified as a serine protease by other authors^47^. The protease gp175 forms a critical step in the maturation of phages, where the protease gp175 cleaves many head proteins, including the major capsid protein and five major structural inner body proteins^47,48^.

In view of these results, phage receptor on *P. aeruginosa* PAR50 was determined among OM proteins by incubating bacteria with PA-PP, followed by extraction of OM proteins and comparing to the OM protein profile extracted from untreated bacteria via SDS-PAGE (Fig. 8). The target protein band that disappeared from treated OM protein samples was identified as a porin protein. Porins are OM proteins (Opr) act as aqueous channels allowing the nonspecific diffusion of small hydrophilic molecules^49^. OprF is the major porin protein in *P. aeruginosa*, highly antigenic and nonspecific to solutes, and allows diffusion of solutes very slow^1,50^. The low permeability of OprF is a major factor that enhances other types of resistance mechanisms and often causes strong multidrug resistance in *P. aeruginosa*^50^. In general, Opr represents substrate-specific transport systems to compensate for the low permeability in *P. aeruginosa* OM and allow the uptake of nutrients which exist at low concentrations in the environment^49^. For instance, OprB and OprB2 for the diffusion of glucose, OprP and OprO facilitate the passage of phosphate, whereas OprD is responsible for the diffusion of basic amino acids and small peptides^49,51^.

Despite significant earlier research focus on phage components and their effects on bacterial cells, insufficient data are available on phage proteins belonging to the serine protease family that are active against porin proteins in OM of bacteria. It has been known that the bacterial lipopolysaccharide was defined as a receptor for PB1 and PB1-like viruses (e.g *Pseudomonas* phage 14-1)^52^, however, these phages encode also several proteins with activity against other bacterial structures i.e. phage 14-1 encodes structural protein gp38, that capable of degrading the peptidoglycan of *P. aeruginosa*^46^. In fact, although the functions of hypothetical proteins of PB1 viruses or PB1-like viruses are still unknown, the coiled coils have been predicted, indicating for protein interactions with other phage or host proteins. The gp8 and gp10 proteins carry both signal peptides and two and three transmembrane domains, respectively, and are clearly targeted to the outer membrane^46^. In another study, 37 host complex-associated phage proteins were identified as hypothetical phage proteins targeting key protein complexes of *P. aeruginosa*. Eight of them showed an inhibitory effect on bacterial growth upon episomal expression, indicating that these phage proteins are potentially involved in hijacking the host complexes. For instance, gp12 protein of *Pseudomonas* phage 14-1, inhibits transcription of *P. aeruginosa* RNA through interacts with the α subunit of RNA polymerase, as well as gp70 protein of *Pseudomonas* phage 14-1, inhibits the growth of *P. aeruginosa* by stopping cells division, followed by cells death^43^.

In this study, phage protein PA-PP was confirmed as an effective antimicrobial agent against *P. aeruginosa* PAR50 without the addition of other factors, although its activity was not determined as bactericidal or bacteriostatic. PA-PP protein was additionally used for synergistic treatment with antibiotics for *P. aeruginosa* PAR50 to evaluate its effect on antibiotic activity. As mentioned above, *P. aeruginosa* PAR50 strain isolated from patients with diabetic foot ulcers shows resistance to several antibiotics, in agreement with previous findings^53–55^. We observed changes in the efficacy of a number of the same antibiotics (piperacillin, ticarcillin-clavulanic acid, ceftriaxone, amikacin, gentamicin and tobramycin) against *P. aeruginosa* PAR50 treated previously with PA-PP protein relative to their efficacy against untreated *P. aeruginosa* PAR50. For instance, the inhibition zone of piperacillin increased in diameter from 14 mm on the plate with untreated bacteria to 22 mm on the plate with treated bacteria, indicating greater efficiency against *P. aeruginosa* PAR50 in combination with PA-PP protein. Consistent with earlier results^30,56,57^, these findings support the ability of phage protein to improve antibiotic efficacy. However, the mechanisms by which phage proteins improve antibiotic efficacy against microorganisms are currently a subject of controversy. Recently, a bacteriophage, OMKO1, of *P. aeruginosa* was shown to utilize the outer membrane porin M (OprM) of multidrug efflux systems, MexAB and MexXY, as a receptor binding site, causing changes in the efflux pump mechanism that plays an important role in resistance, this in turn, increasing sensitivity to several antibiotics^33^. This account is in agreement with our finding that one of OM porins acts as a PA-PP substrate.

In summary, we have classified phage PA-PP protein as a hypothetical protein belongs to serine protease family and demonstrated that its antibacterial activity is related to enzymatic activity on specific bacterial membrane protein targets. Our data provide valuable insights into the mechanisms of action of bacteriophage proteins that may serve as antibacterial agents. Further detailed studies are required to establish the precise functions of this protein and several other phage proteins.

## Materials and Methods

### Bacterial strains

*Pseudomonas aeruginosa* PAR21 and PAR50 strains were isolated from diabetic foot ulcer patients from the Institute of Microbiology, Medical College of Jagiellonian University. The phage host, *P. aeruginosa* PCM 2720, was obtained from the Polish Collection of Microorganisms (PCM) at the Institute of Immunology and Experimental Therapy (Wrocław, Poland).

### Sensitivity test for antibiotics

Both PAR21 and PAR50 strains of *P. aeruginosa* were subjected to several commercial antibiotic disks (piperacillin, piperacillin-tazobactam, ticarcillin-clavulanic acid, ceftazidime, cefepime, cefotaxime, ceftriaxone, imipenem, ciprofloxacin, gentamicin, amikacin, tobramycin and netilmicin) using the disk diffusion susceptibility method described by Bauer *et al*.^58^ and the Clinical and Laboratory Standards Institute CLSI^59^. The zones of growth inhibition around each antibiotic disk were measured to the nearest millimeter, and the zone diameter of each antibiotic compared and interpreted using the criteria published by the CLSI (Supplementary Table S2)^60^.

### Isolation of *P. aeruginosa* exopolysaccharide (EPS)

For preparation of EPS, bacterial cells were cultivated in tryptic soy broth (TSB) (17 g l^−1^ pancreatic digested, 3 g l^−1^ soy bean peptone, 2.5 g l^−1^ KH_2_PO_4_, 5 g l^−1^ NaCl) with 0.7 g l^−1^ glucose at 37 °C for 48 h, followed by centrifugation at 6000 × g and 4 °C for 15 min. The supernatant of bacterial cultures was treated with phenol (final concentration 0.5%) at 4 °C overnight and subjected to centrifugation (9000 × g, 4 °C, 15 min). The supernatant was pooled, precipitated with four volumes of 96% ethanol (−20 °C overnight) and centrifuged (6000 × g, 4 °C, 15 min). The pellet was collected, resuspended in 50 ml of 1 M NaCl, mixed and re-precipitated under the same conditions. The pellet was harvested via centrifugation at 6000 × g and 4 °C for 15 min, mixed with 5 ml PBS, digested with proteinase K (37 °C for 1 h), dialyzed against distilled water (4 °C for 48 h) and lyophilized^61^.

### Isolation of *P. aeruginosa* outer membrane proteins

Outer membrane proteins were isolated from *P. aeruginosa* PAR21 and PAR50 strains using the sodium dodecyl sulfate (SDS) extraction technique described by Mizuno and Kageyama^62^. Bacterial strains were grown in nutrient broth (peptone 10 g l^−1^, yeast extract 10 g l^−1^, NaCl 5 g l^−1^) with shaking at 37 °C. Cells were harvested at the late log phase and disrupted via sonication in 10 mm sodium phosphate buffer (pH 7.2) using a Branson sonifier at 4 °C. Cell envelopes were pooled via differential centrifugation at 100,000 × g for 60 min, washed twice with the same buffer and lyophilized. Next, lyophilized cell envelopes were treated with SDS solution (2% SDS-10% glycerol-10 mM Tris-HCl, pH 7.8) at 30 °C for 60 min, followed by ultracentrifugation at 100,000 × g for 60 min. The combined soluble fraction (fraction A) mainly composed of proteins was re-pooled under the same conditions. Insoluble fraction B was treated with SDS solution supplemented with 0.1 M NaCl at 30 °C for 60 min and centrifuged at 100,000 × g for 60 min at 25 °C, and the supernatant (fraction C) mainly comprising proteins collected. The remaining pellet (fraction D) predominantly consisting of peptidoglycan and proteins was washed twice with distilled water and dissolved in 300 ml Triton X-100-urea buffer (2% Triton X-100-6 M urea-10 mM Tris-HCl, pH 7.4) at 40 °C for 60 min, followed by centrifugation at 100,000 × g for 60 min at 25 °C to obtain the supernatant. The extraction was repeated once more under the same conditions. Proteins in the pooled supernatant were precipitated by adding two volumes of cold acetone, washed once with 90% cold acetone, dissolved in a small volume of Triton X-100-urea buffer and dialyzed against the same buffer at room temperature overnight.

### Isolation and cultivation of bacteriophages

The phage used in this study was isolated from Wroclaw sewage. Lysogeny broth (LB) medium (10 g l^−1^ tryptone, 5 g l^−1^ yeast extract, 5 g l^−1^ NaCl) was employed for phage cultivation with *P. aeruginosa* PCM 2720 as the phage host. Bacterial cells were infected with 1 × 10^9^ pfu/ml phage at an optical density (OD)_600_ of 0.6 and incubated at 37 °C with shaking (120–150 rpm) until visual clearance. Subsequently, cells were killed by adding 1% (v/v) chloroform and incubation with shaking for 30 min at room temperature, followed by removal of bacterial debris via centrifugation at 6000 × g and 4 °C for 15 min, and finally, sterilization of supernatant using bacterial membrane filters (0.22 μm). Phage solution was kept at 4 °C as a source of phage and its crude enzyme^63^.

### Determination of phage activity via spot assay

Bacterial cells activated previously in LB were transferred (1 ml at OD_600_ 0.1–0.3) onto an agar plate, spread on top and left to dry. The phage was pipetted (10 µl) onto the agar top and left to dry, followed by 18–24 h incubation at 37 °C. Plates were duplicated for each bacterial strain^64^.

### Determination of routine test dilution (RTD) and phage titration

The routine test dilution (RTD) was obtained using a spot assay whereby the phage was diluted in PBS to generate serial concentrations from 10^−1^ to 10^−10^. The diluted phage (10 µl) was applied on a lawn of bacterial cells and the highest dilution producing complete lysis taken as the routine test dilution^65^. The phage titer was determined with the plaque assay described by Adams^66^. Briefly, serial dilutions (10^−1^ to 10^−10^) of phage were prepared in PBS. An aliquot of host culture (200 μl) at OD_600_ of 0.2–0.3 was added to the soft agar overlay tube (melted and subsequently cooled to 45–50 °C), followed by 200 μl diluted phage. The mixture was vortexed and immediately poured onto the agar plate. This step was repeated with all phage dilutions. Dishes were rotated gently upright on a flat surface for even distribution of the mixtures, left to solidify and incubated at 37 °C for 18–20 h (59). Phage titers were calculated by counting the plaques that indicate patches of dead bacteria, with each plaque representing a single virus.

### Isolation and Purification of PA-PP protein

PA-PP protein was isolated from phage particles using the acid method described by Rieger *et al*.^67^. Phage (1 × 10^9^ pfu/ml) was fragmented by decreasing pH to 3.5 using 0.1 N HCl at 37 °C for 20 min, followed by neutralization with 0.2 M aqueous Tris buffer and digestion by adding DNase I and Mg^2+^ (final concentrations of 20 µg/ml and 2 mM, respectively) and incubation at 37 °C for 3 h. Isolated protein was concentrated via ultrafiltration using amicon ultra centrifugal filter (Millipore, Billerica, USA) with molecular weight cutoffs of 10 kDa and subsequently 30 kDa at 4000 × g and 4 °C. PA-PP protein was purified on a Toyopearl HW-55S column (1.6 × 100 cm, Tosoh Bioscience LLC) using 0.06 M phosphate buffer, pH 7, for equilibration at a flow rate at 0.5 ml/min. The procedure was performed using the FPLC system (Amersham Pharmacia Biotech). Fractions of interest were selected based on activity against *P. aeruginosa* PAR50 via the spot assay method. The protein concentration was estimated using the bicinchoninic acid (BCA) assay^68^ with BCA protein assay kit (Thermo, Rockford, USA). Molecular mass was determined via SDS-PAGE.

### SDS-PAGE of PA-PP

SDS-PAGE was performed according to the protocol of Laemmli^69^. Briefly, the protein was mixed with sample buffer (0.075 M Tris–HCl, pH 6.8, 2% SDS, 10% glycerol, 5% 2-mercaptoethanol and bromophenol blue) and heated in a water bath at 95 °C for 5 min. A broad-range protein ladder (Thermo, Rockford, USA) was employed as the standard to determine the molecular mass of protein. Protein separation was achieved via a 12.5% polyacrylamide gel prepared with resolving buffer (1.5 M Tris–HCl, pH 8.8, 0.4% SDS, 100 ml H_2_O), 10% ammonium persulfate and 30% acrylamide solution. Electrophoresis was achieved in running buffer (0.025 M Tris, 0.192 M glycine, pH 8.3, H_2_O). Protein bands were visualized by staining the gel with CBB-R250 (1% CBB, 10% acetic acid, 50% methanol, 40% H_2_O).

### Identification of PA-PP protein

The purified protein was subjected to electrophoresis using 10% polyacrylamide gels, one with Laemmli conditions^69^ and another under non-reducing conditions (without 2-mercaptoethanol and heat). The target protein band was excised from the gel and digested with trypsin. Peptides mixture was separated using liquid chromatography and mass spectrometry, and the mass peptide fragments were measured by mass spectrometer LC-MS/MS Orbitrap. Peptide molecular masses were compared with the protein sequence database (NCBI/UniProt database) using MS/MS ion search of the Mascot search engine (Matrix Science, London, UK, http://www.matrixscience.com/) and statistical analysis.

### Spot assay

PA-PP activity against *P. aeruginosa* PAR50 was determined using spot assay as described above, where aliquots of protein (10 µl) were pipetted onto agar plates containing a lawn of *P. aeruginosa* PAR50 and incubated at 37 °C for 18 h.

### Zymography

PA-PP activity was also determined using the renaturing gel electrophoresis technique described by Foster^70^. Two 12.5% (w/v) polyacrylamide gels were used for each strain, whereby one was mixed with 0.1% (w/v) bacterial cells and another with bacterial EPS. The PA-PP protein was prepared under non-reducing conditions (without boiling or 2-mercaptoethanol) and loaded on each gel. Electrophoresis was achieved in running buffer at 20 mA. Each gel was rinsed for 30 min in 250 ml distilled water at room temperature with gentle agitation and renatured (3 × 30 min) in 250 ml renaturation buffer (1% (v/v) Triton X-100 (Sigma-Aldrich), 20 mM MgCI_2_, 25 mM Tris-HCl, pH 7.5) at room temperature with gentle agitation. Gels were transferred to 250 ml of the same buffer and incubated for 16 h at 37 °C with gentle agitation. After incubation, gels were washed in distilled water and visualized using the protocol of García-Carreño *et al*.^71^ with a slight modification in that staining was performed using CBB-R250 (1% CBB, 10% acetic acid, 50% methanol and 40% H_2_O).

### Spectrophotometric assay

PA-PP protein efficacy against *P. aeruginosa* PAR21 and PAR50 strains was evaluated by an automated spectrophotometer (Tecan Spark 10 M multimode reader) to monitor changes in bacterial density (OD_600_) in a mixture of bacterial cells and phage protein. Flat-bottomed 96-well MaxiSorp plates (Nunc) were coated with 200 µl bacterial suspension grown to OD_600_ of 0.2 and 50 µl diluted protein (100, 50, 25, and 12.5 µg/ml) and incubated at 37 °C with continuous shaking. Absorbance was measured at 600 nm automatically every hour for 7 h and the resulting OD for each well was recorded by SparkControl Magellan and then imported to Excel (Microsoft) for further analysis. Bacteria with PBS were used as a control. Three replicate experiments were performed for each protein dilution.

### Assay of enzymatic activity

The ability of PA-PP protein to digest bacterial EPS was tested via the Nelson-Samogyi method for reducing sugar^72^. EPS (50 µl; 1 mg/ml PBS) was mixed with 100 µl PA-PP protein and incubated at 37 °C overnight on a rotary shaker. The total volume was made up to 2 ml with distilled water, followed by the addition of 1 ml alkaline copper tartrate solution to each tube and heating in a bath of boiling water for 10 min. After cooling, 1 ml arsenomolybdic acid was added to all the tubes and the volume made up to 10 ml with water. Absorbance of blue color was read at 620 nm after 10 min on a microplate spectrophotometer (PowerWave HT, Biotek). Hydrolysis of EPS by 10 M HCl was used as a positive control, EPS with PBS as a negative control and distilled water as the blank. Serial dilutions of glucose and galactose were used as the standard.

### Assay of proteolytic activity

Resorufin-labeled casein (Roche, Mannheim, Germany) was utilized as the substrate to determine the proteolytic activity of PA-PP according to the procedure of Twining^73^. Substrate solution (50 µl; 0.4% w/v resorufin-labeled casein in double-distilled water) was mixed with 50 µl incubation buffer (0.2 M Tris, pH 7.8, 0.02 M CaCl_2_) and 100 µl PA-PP protein for 30 min at 37 °C, followed by 480 µl stop reagent (5% w/v TCA). Next, the solution was incubated for 10 min at 37 °C and subsequently centrifuged for 5 min. The supernatant fraction (400 µl) was mixed with 600 µl assay buffer (0.5 M Tris–HCl, pH 8.8) and absorbance at 574 nm immediately read against the blank on a microplate spectrophotometer. Casein with trypsin or PBS was used as the positive and negative control, respectively.

### Scanning electron microscopy at low voltage (LV-SEM)

*P. aeruginosa* PAR21 and PAR50 strains were incubated on agar plates at 37 °C for 10 h and then 20 µl (250 µg/ml) PA-PP protein was applied topically on some colonies on the plate. As a control, PBS was applied to a different location on the same dish and incubation continued at 37 °C overnight. Silicon chips (7 × 7 mm) were pressed against the targeted site on the plate to attach bacteria on the chip surface. The bacteria-containing chip was rinsed in PBS at room temperature and immersed in 2.5% glutaraldehyde in 0.1 M cacodylate buffer. Fixation was continued for 30 min, followed by washing (5 × 30 min, 4 °C) with 0.1 M cacodylate buffer and dehydration in serial concentrations of ice-cold methanol (25%, 40%, 60%, 80% and 100%). All samples designed for imaging at room temperature underwent critical point drying with 100% methanol exchanged for liquid CO_2_ in an automated manner (CPD300 AUTO, Leica Microsystems, Vienna, Austria) and were imaged under a cross-beam scanning electron microscope equipped with a Schottky field-emission cathode (Auriga 60, Carl Zeiss, Oberkochen, Germany) at 1.2 kV accelerating voltage.

### Detection of phage receptor on the bacterial cell surface

Phage receptor on bacterial cell surfaces was detected in both EPS and OM proteins. EPS was isolated and subjected to PA-PP as described above. Detection of the receptor in OM proteins was achieved by treatment of each bacterial strain at OD_600_ of 0.1 with 100 µl PA-PP protein (250 µg/ml), followed by vigorous shaking at 37 °C for 18–24 h. Untreated culture was used as the control. Cells were harvested via centrifugation at 6000 × g for 15 min at 4 °C and dried via lyophilization. Lyophilized cells were suspended in 0.01 M N-2-hydroxyethylpiperazine-Nʹ-2-ethanesulfonic acid (HEPES), pH 7.4, and disrupted via sonication (Braunsonic sonifier), followed by removal of debris via centrifugation at 6000 × g for 10 min at 4 °C. OM proteins were extracted using SDS extraction method as described above. The contents of proteins isolated from both treated and untreated bacterial cells were compared using SDS-PADE. The target protein band was identified via LC-MS/MS and comparison of peptide masses with protein sequences (NCBI/UniProt database).

### Evaluation of the impact of PA-PP protein on antibiotic activity

Bacterial cells grown to OD_600_ of 0.1 were mixed with 50 µl PA-PP protein, incubated for 1 h at 37 °C and evenly spread onto an agar plate, followed by distribution of antibiotic disks (piperacillin, ticarcillin-clavulanic acid, gentamicin, amikacin, and tobramycin) on the agar surface and incubation for 18–24 h at 37 °C. Agar plates with untreated bacteria were used as the control.

### Statistical analysis

Data were analyzed with the t test and one-way ANOVA to compare differences between two or more groups. The results were expressed as means ± SEM of three independent measurements and considered significant at *P* values < 0.05. Statistical analysis was performed using GraphPad Prism 8 (GraphPad Software, USA).

### Ethical statement

We confirm that all experiments in this work didn’t perform on humans and/or the use of human tissue samples, and animals.

## Supplementary information

Supplementary Data
