# Supplementary material for: Identification and characterization of phage protein and its activity against two strains of multidrug-resistant Pseudomonas aeruginosa
Source: Sci Rep. 2019 Sep 17;9:13487. doi: 10.1038/s41598-019-50030-5 (PMC6748951; doi:10.1038/s41598-019-50030-5)
Supplement: Supplementary file 1 — Supplementary Data [file 41598_2019_50030_MOESM1_ESM.pdf]

Supplementary Data

Identification and characterization of phage protein and its activity against two strains of multidrug-resistant *Pseudomonas aeruginosa*

Fairoz Al-Wrafy[1,2*], Ewa Brzozowska[2*], Sabina Górska[2], Marek Drab[2], Magdalena Strus[3], Andrzej Gamian[2]

[1] Department of Applied Microbiology, Faculty of Applied Science, Taiz University, 6350 Taiz, Yemen.

[2] Hirszfeld Institute of Immunology and Experimental Therapy, Polish Academy of Sciences, 53-114 Wrocław, Poland.

[3] Department of Microbiology, Medical College, Jagiellonian University, 31-007 Krakow, Poland.

[*] Correspondence:

Fairoz Al-Wrafy: f_alwrafy@yahoo.com

Ewa Brzozowska: ewa.brzozowska@hirszfeld.pl

Antimicrobial agent	Disk content	Zone diameter, nearest whole [mm]			
		PAR21		PAR50	
Piperacillin	100 µg	28	S	14	R
Piperacillin- tazobactam	100/10 µg	28	S	25	S
Ticarcillin-clavulanic acid	75/10 µg	19	I	14	R
Ceftazidime	30 µg	27	S	25	S
Cefepime	30 µg	18	S	17	S
Cefotaxime	30 µg	20	I	22	I
Ceftriaxone	30 µg	< 13	R	< 13	R
Imipenem	10 µg	24	S	30	S
Ciprofloxacin	5 µg	32	S	35	S
Gentamicin	10 µg	17	S	<12	R
Amikacin	30 µg	20	S	14	R
Tobramycin	10 µg	18	S	<12	R
Netilmicin	30 µg	20	S	20	S

Table S1. Diameters of inhibition zones of different antibiotics against *P. aeruginosa* PAR21 and PAR50. R. Resistant, I. Intermediate and S. Susceptible

Antimicrobial agent	Disk content	Zone diameter, nearest whole [mm]		
		R	I	S
Piperacillin	100 µg	≤ 14	15-20	≥21
Piperacillin- tazobactam	100/10 µg	≤ 14	15-20	≥21
Ticarcillin-clavulanic acid	75/10 µg	≤ 15	16-23	≥24
Ceftazidime	30 µg	≤ 14	15-17	≥ 18
Cefepime	30 µg	≤ 14	15-17	≥ 18
Cefotaxime	30 µg	≤ 14	15-22	≥ 23
Ceftriaxone	30 µg	≤13	14-20	≥ 21
Imipenm	10 µg	≤ 13	14-15	≥16
Ciprofloxacin	5 µg	≤15	16-20	≥21
Gentamicin	10 µg	≤12	13-14	≥15
Amikacin	30 µg	≤ 14	15-16	≥17
Tobramycin	10 µg	≤12	13-14	≥15
Netilmicin	30 µg	≤12	13-14	≥15

Table S2. The inhibition zone diameter for each antibiotic according to the criteria published by the Clinical and Laboratory Standards Institute (CLSI)[1] . **R**. Resistant, **I**. Intermediate and **S**. Susceptible

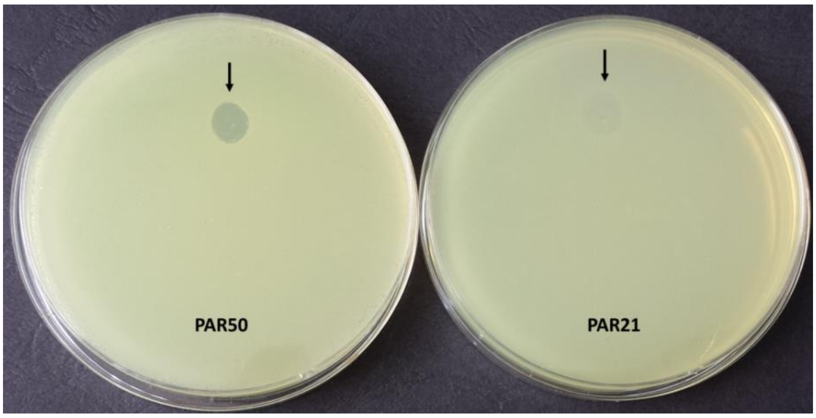

Figure S1. Spot assay using 10 µl phage sample applied on a lawn of *P. aeruginosa* strains. Translucent clearance at the spot area was observed against PAR50, but not PAR21.

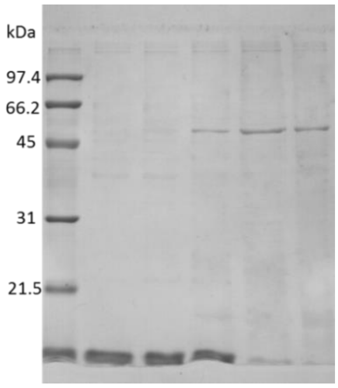

Figure S2. Full-length uncropped gel used in figure 1.

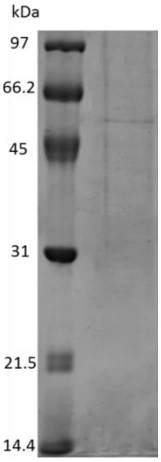

Figure S3. Full-length gel used in figure 2.

```
  1 MISQSRYIRI ISGVGAGAPV AGRKLILRVM TTNNVIPPGI VIEFDNANAV
 51 LSYFGAQSEE YQRAAAYFKF ISKSVNSPSS ISFARWVNTA IAPMVVGDNL
101 PKTIADFAGF SAGVLTIMVG AAEQNITAID TSAATSMDNV ASIIQTEIRK
151 NADPQLAQAT VTWNQNTNQF TLVGATIGTG VLAVAKSADP QDMSTALGWS
201 TSNVVNVAGQ AADLPDAAVA KSTNVSNNFG SFLFAGAPLD NDQIKAVSAW
251 NAAQNNQFIY TVATSLANLG TLFTLVNGNA GTALNVLSAT AANDFVEQCP
301 SEILAATNYD EPGASQNYMY YQFPGRNITV SDDTVANTVD KSRGNYIGVT
351 QANGQQLAFY QRGILCGGPT DAVDMNVYAN EIWLKSAIAQ ALLDLFLNVN
401 AVPASSTGEA MTLAVLQPVL DKATANGTFT YGKEISAVQQ QYITQVTGDR
451 RAWRQVQTLG CWINITFSSY TNSNTGLTEW KANYTLIYSK GDAIRFVEGS
501 DVMI
```

Figure S4. Identification of PA-PP1 protein using mass spectrometer LC-MS/MS. The protein sequence, matched peptides are shown in bold red.

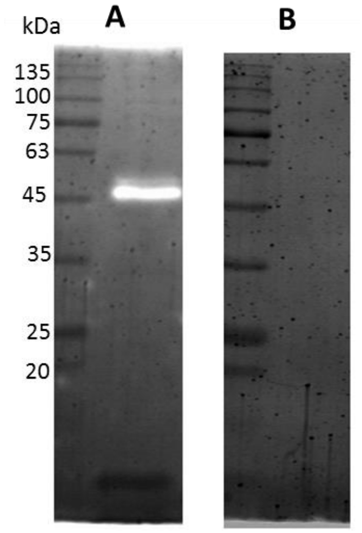

Figure S5. Full-length gel used in figure **3**

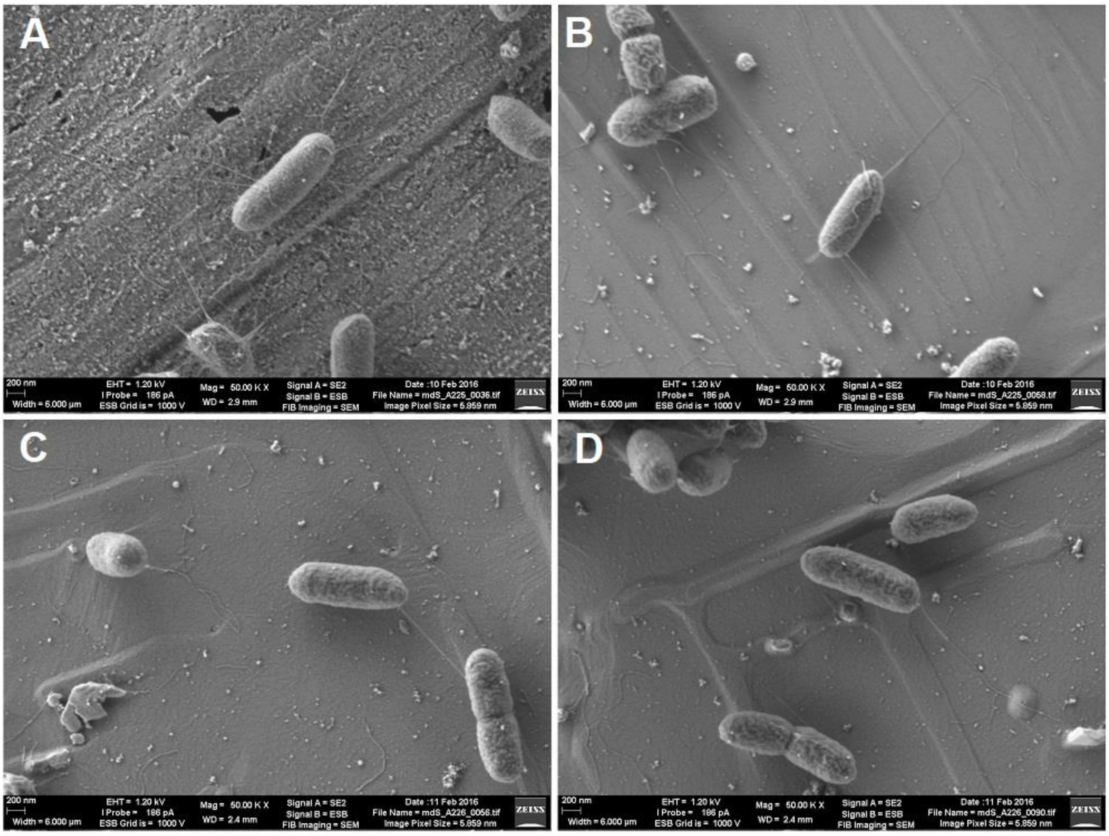

Figure S6. Low voltage scanning electron microscopy (LV-SEM) to determine the effects of PA-PP on *P. aeruginosa* PAR21. A and B represent intact cells not exposed to protein. C and D represent PAR21 cells exposed to PA-PP, which appear similar to the non-treated cells showing no defects.

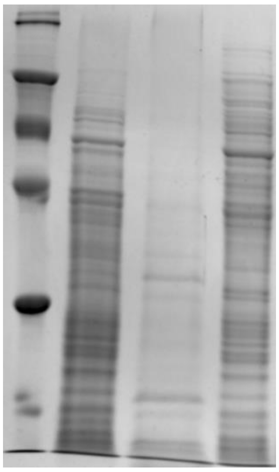

Figure S7. Full-length gel used in figure 8.

```
  1 MKLKNTLGVV IGSLVAASAM NAFAQGQNSV EIEAFGKRYF TDSVRNMKNA
 51 DLYGGSIGYF LTDDVELALS YGEYHDVRGT YETGNKKVHG NLTSLDAIYH
101 FGTPGVGLRP YVSAGLAHQN ITNINSDSQG RQQMTMANIG AGLKYYFTEN
151 FFAKASLDGQ YGLEKRDNGH QGEWMAGLGV GFNFGGSKAA PAPEPVADVC
201 SDSDNDGVCD NVDKCPDTPA NVTVDANGCP AVAEVVRVQL DVKFDFDKSK
251 VKENSYADIK NLADFMKQYP STSTTVEGHT DSVGTDAYNQ KLSERRANAV
301 RDVLVNEYGV EGGRVNAVGY GESRPVADNA TAEGRAINRR VEAEVEAEAK
```

Figure S8. Identification of bacterial OM protein which was targeted by PA-PP protein mass spectrometer LC-MS/MS. The protein sequence obtained from the mass spectrometer LC-MS/MS, matched peptides are shown in bold red.

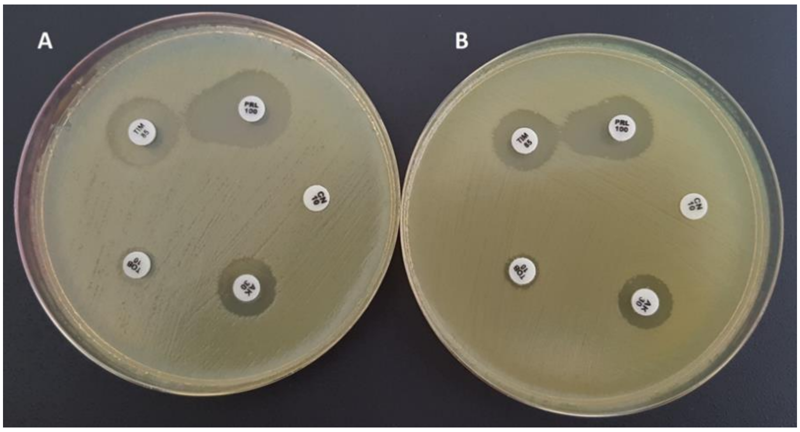

Figure S9. Synergistic activity of antibiotics and PA-PP. **A**. Antibiotics were applied to *P. aeruginosa* PAR50 treated with PA-PP protein. **B**. Antibiotics were applied to untreated *P. aeruginosa* PAR50.

1- Clinical and Laboratory Standards Institute (CLSI). Performance standards for antimicrobial susceptibility testing. Informational supplement M100–S24, 24th ed. 34, 58–60. (Clinical and Laboratory Standards Institute, Wayne, PA. USA. 2014).
